# Targeting Fn14 as a therapeutic target for cachexia reprograms the glycolytic pathway in tumour and brain in mice

**DOI:** 10.1007/s00259-024-06836-1

**Published:** 2024-07-26

**Authors:** Ingrid Julienne Georgette Burvenich, Laura Danielle Osellame, Angela Rigopoulos, Nhi Huynh, Zhipeng Cao, Nicholas Johannes Hoogenraad, Andrew Mark Scott

**Affiliations:** 1 Tumour Targeting Laboratory, Olivia Newton-John Cancer Research Institute, 145 Studley Road, Melbourne, Heidelberg, VIC 3084 Australia; 2 School of Cancer Medicine, La Trobe University, Melbourne, VIC 3086 Australia; 3 Department of Biochemistry and Genetics, La Trobe Institute for Molecular Science, La Trobe University, Melbourne, VIC 3083 Australia; 4 Department of Molecular Imaging and Therapy, Austin Health, Melbourne, VIC 3083 Australia; 5 Department of Medicine, University of Melbourne, Melbourne, VIC 3052 Australia

**Keywords:** Fn14 receptor, TWEAK, [^18^F]FDG PET, Cancer cachexia, 002 antibody

## Abstract

**Purpose:**

Cachexia is a complex syndrome characterized by unintentional weight loss, progressive muscle wasting and loss of appetite. Anti-Fn14 antibody (mAb 002) targets the TWEAK receptor (Fn14) in murine models of cancer cachexia and can extend the lifespan of mice by restoring the body weight of mice. Here, we investigated glucose metabolic changes in murine models of cachexia via [^18^F]FDG PET imaging, to explore whether Fn14 plays a role in the metabolic changes that occur during cancer cachexia.

**Methods:**

[^18^F]FDG PET/MRI imaging was performed in cachexia-inducing tumour models versus models that do not induce cachexia. SUV_average_ was calculated for all tumours via volume of interest (VOI) analysis of PET/MRI overlay images using PMOD software.

**Results:**

[^18^F]FDG PET imaging demonstrated increased tumour and brain uptake in cachectic versus non-cachectic tumour-bearing mice. Therapy with mAb 002 was able to reduce [^18^F]FDG uptake in tumours (*P* < 0.05, *n* = 3). Fn14 KO tumours did not induce body weight loss and did not show an increase in [^18^F]FDG tumour and brain uptake over time. In non-cachectic mice bearing Fn14 KO tumours, [^18^F]FDG tumour uptake was significantly lower (*P* < 0.01) than in cachectic mice bearing Fn14 WT counterparts. As a by-product of glucose metabolism, l-lactate production was also increased in cachexia-inducing tumours expressing Fn14.

**Conclusion:**

Our results demonstrate that Fn14 receptor activation is linked to glucose metabolism of cachexia-inducing tumours.

**Graphical Abstract:**

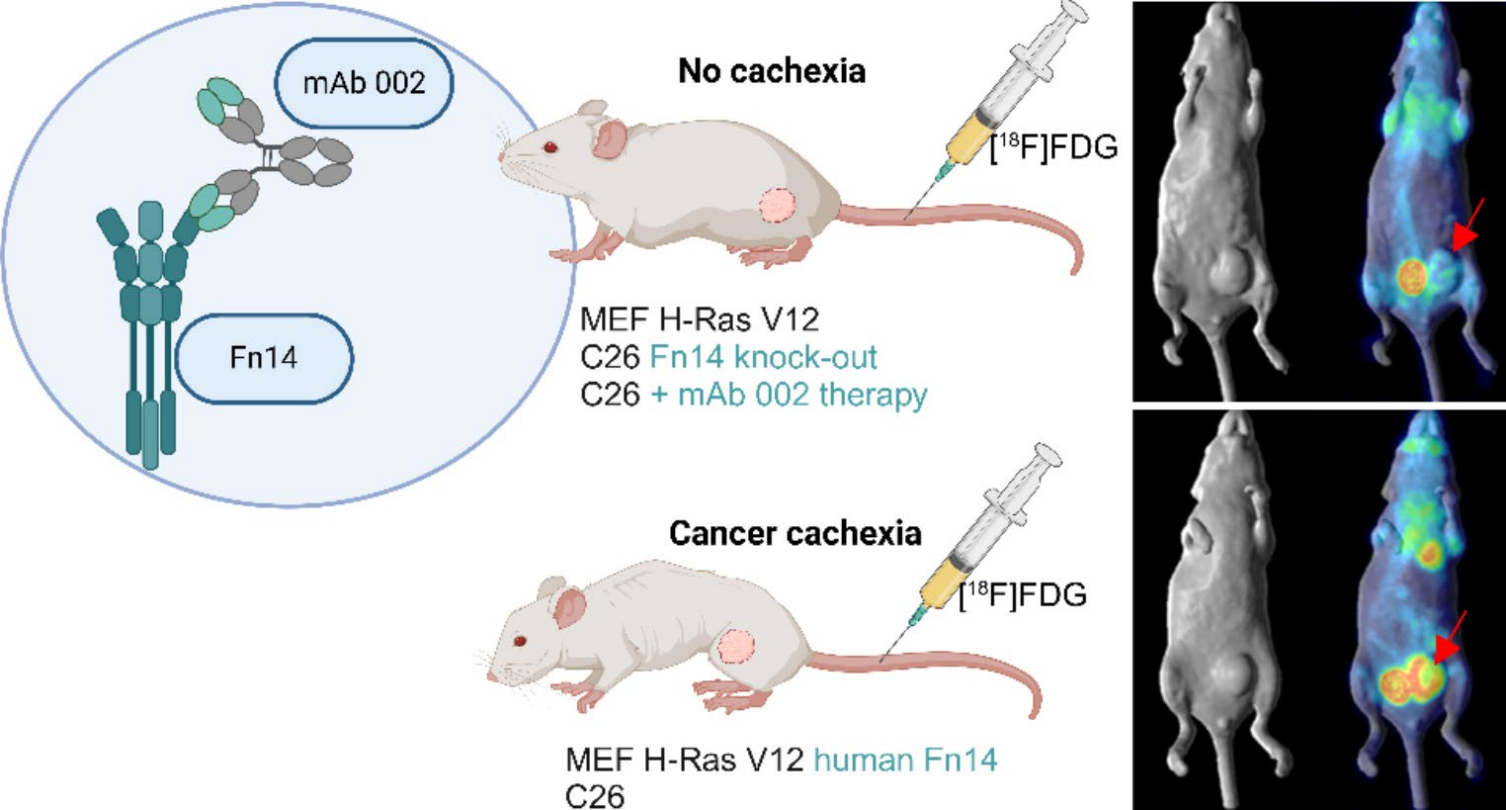

**Supplementary Information:**

The online version contains supplementary material available at 10.1007/s00259-024-06836-1.

## Introduction

Cachexia is a severe wasting syndrome that is often observed in late to terminal stages of many chronic illnesses, affecting up to 80% of cancer patients [1, 2]. It is well documented that cachexia cannot be alleviated by an increase in appetite or caloric intake [3]. In traditional starvation or *anorexia nervosa*, weight loss is primarily from fat loss, with wasting in cachexia being due to both adipose tissue and skeletal muscle loss [4]. Patients with cancer cachexia display severe fatigue together with a reduced quality of life [5]. As cachexia limits the ability of patients to respond to and tolerate anti-cancer therapy, it is frequently referred to as “the last illness” with the wasting syndrome often responsible for death rather than the original condition [6]. Despite the central importance of cancer cachexia for tumour disease outcome, there is still no defined standard of care to effectively counteract progressive wasting and there is no FDA approved treatment for this condition [7].

Fn14 (Fibroblast growth factor-inducible immediate-early response protein 14) is a plasma membrane protein and is activated by TWEAK (TNF-like weak inducer of apoptosis) [8], and is involved in wound healing [9], angiogenesis [10], proliferation [11], apoptosis [12] and inflammation [13, 14]. When Fn14 is expressed on tumour cells, it triggers the onset of cachexia [15] independent of its ligand, TWEAK [15]. This suggests that Fn14 on the tumour membrane utilizes a divergent activation mechanism which drives an uncharacterized cachexia specific signalling nexus, and not the known TWEAK/Fn14 pathway. To evaluate the role of Fn14 in cachexia, a highly specific monoclonal antibody (mAb 002) was developed that reverses cancer cachexia driven by Fn14 [15]. MAb 002 has shown promise in preclinical models of cancer cachexia, blocking fat and muscle wasting, without inhibiting tumour growth [15]; although the anti-Fn14 antibody can induce tumour growth inhibition in some tumour models (unpublished data).

Cancer cells have a distinctive ability to “rewrite” their metabolism, emphasizing the metabolism of glucose to lactate [16]. The glycolytic pathway generates 2–4 adenosine triphosphate (ATP) molecules per molecule of glucose, compared with 36 ATP when one molecule of glucose is completely oxidized via oxidative phosphorylation in mitochondria [17]. ^18^F-fluorodeoxyglucose ([^18^F]FDG) PET is routinely used in the clinical management of cancers, and its utility is thought to be due to enhanced aerobic glycolysis in tumors (the Warburg effect) [18, 19]. As cancer cachexia is a metabolic condition, the metabolic changes occurring in cachectic versus non-cachectic patients may be monitored by whole body molecular imaging with [^18^F]FDG. Indeed, Penet et al., have shown an increase in tumour uptake of [^18^F]FDG in a cachexia inducing murine adenocarcinoma (MAC16) tumour model compared to the histologically similar but non-cachexia-inducing MAC13 tumour model [20]. More recently, Olaechea et al. (2022) demonstrated a significant positive association between significant pretreatment cancer-associated weight loss and primary tumour SUV_Max_. This study showed that [^18^F]FDG uptake in the primary tumour is associated with cancer-associated weight loss in non-small cell lung cancer (NSCLC) and with poor outcome [21].

To explore whether Fn14 plays a role in the metabolic changes occurring during cancer cachexia, we investigated glucose metabolic changes, via [^18^F]FDG PET imaging, in mouse models of cancer cachexia. [^18^F]FDG PET imaging was performed in non-cachectic MEF H-Ras V12 (MEF) versus cachectic MEF H-Ras V12 tumour-bearing NOD scid gamma (NSG) mice expressing human Fn14 (MEF H-Ras V12 hFn14 (MEF Fn14)). Secondly, [^18^F]FDG PET imaging was performed in cachectic C26 colon tumour-bearing mice treated with anti-Fn14 mAb 002 antibody (10 mg/kg) versus vehicle control treated mice. In a final model, [^18^F]FDG imaging was compared in C26 tumours and an Fn14 knock-out variant of the cachexia-inducing C26 tumour model (C26 Fn14 KO). Our results suggest a direct link between Fn14, and [^18^F]FDG uptake and cachexia as well as mechanistic insight into Fn14-driven cachexia.

## Materials and methods

### Cell lines and 002 antibody

Oncogenic murine embryo fibroblast lines, MEF H-Ras V12 (MEF), MEF H-Ras V12 hFn14 (MEF Fn14) and the C26 Fn14 KO mouse colon tumour line have been described previously [15]. Colon-26 murine colon carcinoma cell line (C26) was obtained from CLS Cell Lines Service (Eppelheim, Germany). Cells were cultured in DMEM: F12 (MEF) and RPMI (C26) containing 10% fetal calf’s serum, 1% L-Glutamax and 1% penicillin/streptomycin and incubated at 37 °C in 10% CO_2_.

### Animal model

All models utilized 6-8-week-old NOD scid gamma female mice (Austin Bioresource Facility, VIC, Australia; or Animal Resources Centre, WA, Australia). Body weight and tumour volume were monitored regularly. Tumour volume (TV) was calculated by the formula (length × width^2^)/2, where length was the longest axis and width the measurement at right angles to length. After injection of tumour cells, body weight was monitored every two days until body weight loss was identified (≥ 5% of maximum body weight) after which body weight was monitored daily until the study end-point (body weight loss ≥ 15% of maximum body weight). All animal studies were conducted in compliance with the Australian Code of Practice for the care and use of animals for scientific purposes and were approved by the Austin Health Animal Ethics Committee.

To establish tumours, MEF or MEF Fn14 cells (5 *×* 10^6^ cells*)* in phosphate-buffered saline (PBS) were injected subcutaneously into the left abdominal flank of 6-week-old NSG mice. C26 cells (1 *×* 10^6^) or C26 Fn14 KO (2 *×* 10^6^) in PBS were injected subcutaneously into the left abdominal flank of 6 to 7-week-old NSG mice.

### Therapy studies

C26 tumour-bearing mice were treated with mAb 002 at 10 mg/kg doses (*n* = 5) or PBS vehicle control (*n* = 5) in 100 µL. Mab 002 was administered intraperitoneally on day 7 and day 10 after cell injection.

### [^18^F]FDG PET imaging

All animals were fasted for 3 h prior to i.v. injection of 14.8 MBq [^18^F]FDG whilst still awake. Immediately after injection, mice were anaesthetised by isofluorane inhalation for 1 h prior to PET acquisition and kept warm on a heat pad. Mice were then placed in a warmed imaging chamber for 15 min to allow static acquisition imaging using a dedicated small animal nanoPET/MR camera (nanoScan^®^, Mediso, Budapest, Hungary).

Whole-body in vivo [^18^F]FDG PET imaging of MEF (*n* = 2) or MEF Fn14 (*n* = 2) tumour-bearing mice was performed at the onset of cachexia on day 7, and at endpoint (day 9 following tumour cell injection). In the C26 therapy model, animals treated with 002 (*n* = 3) or vehicle control (PBS) (*n* = 3) were imaged on day 8 and on day 14 following tumour cell injection. In the study comparing C26 Fn14 wildtype versus C26 Fn14 KO tumours, imaging was performed on day 11, day 13 and on day 15 when mice had not reached ethical endpoint.

The multimodality image datasets were archived from the Mediso hybrid imaging systems to a PACS server via DICOM transfer. The co-registration of multi-modality scans, markup of volumes of interest (VOIs) and quantitative image analysis were performed using PMOD 3.8 (PMOD Technologies LLC, Zurich, Switzerland).

The PET images were converted in the units of the standardised uptake value (SUV) which is defined as:

$$SUV\, = \,{{{C_t}(kBq/mL)} \over {{{Injected\,Dose\,(kBq)} \over {Body\,Mass\,(g)}}}}$$

where *C*_*t*_ is the radioactivity concentration in a specific image pixel at time *t* (h) after injection, and injected dose is corrected for the ^18^F decay half-life (109.77 min).

### L-lactate assay

Intratumorally l-lactate levels were measured using an l-lacate assay kit (Abcam; ab65331) according to manufacturer’s instructions with some modifications. Briefly, L-lactate was extracted from 10 mg of material from MEF, MEF Fn14, C26 Fn14 wildtype and C26 Fn14 KO tumours (tumours were taken from mice subjected to imaging studies). Protein levels from each tumour sample were normalized prior to beginning of the assay, and 10 µg of total extract was used for assay development. L-lactate levels were post-normalised to protein amount and expressed as nmol of lactate per mg of tumour.

### Statistical analysis

An unpaired two-tailed t-test was used to determine significant differences between tumour volume (mm^3^), body weight (percentage of maximum body weight), SUV uptake and l-lactate results. Analyses were conducted with Prism 10.0 (GraphPad Software, San Diego, CA). Data are presented as the mean ± SD. L-lactate data is shown as mean ± SEM.

## RESULTS

### [^18^F]FDG PET imaging demonstrates increased glucose uptake over time in tumours of cachectic versus non-cachectic mice

**Fig. 1:**
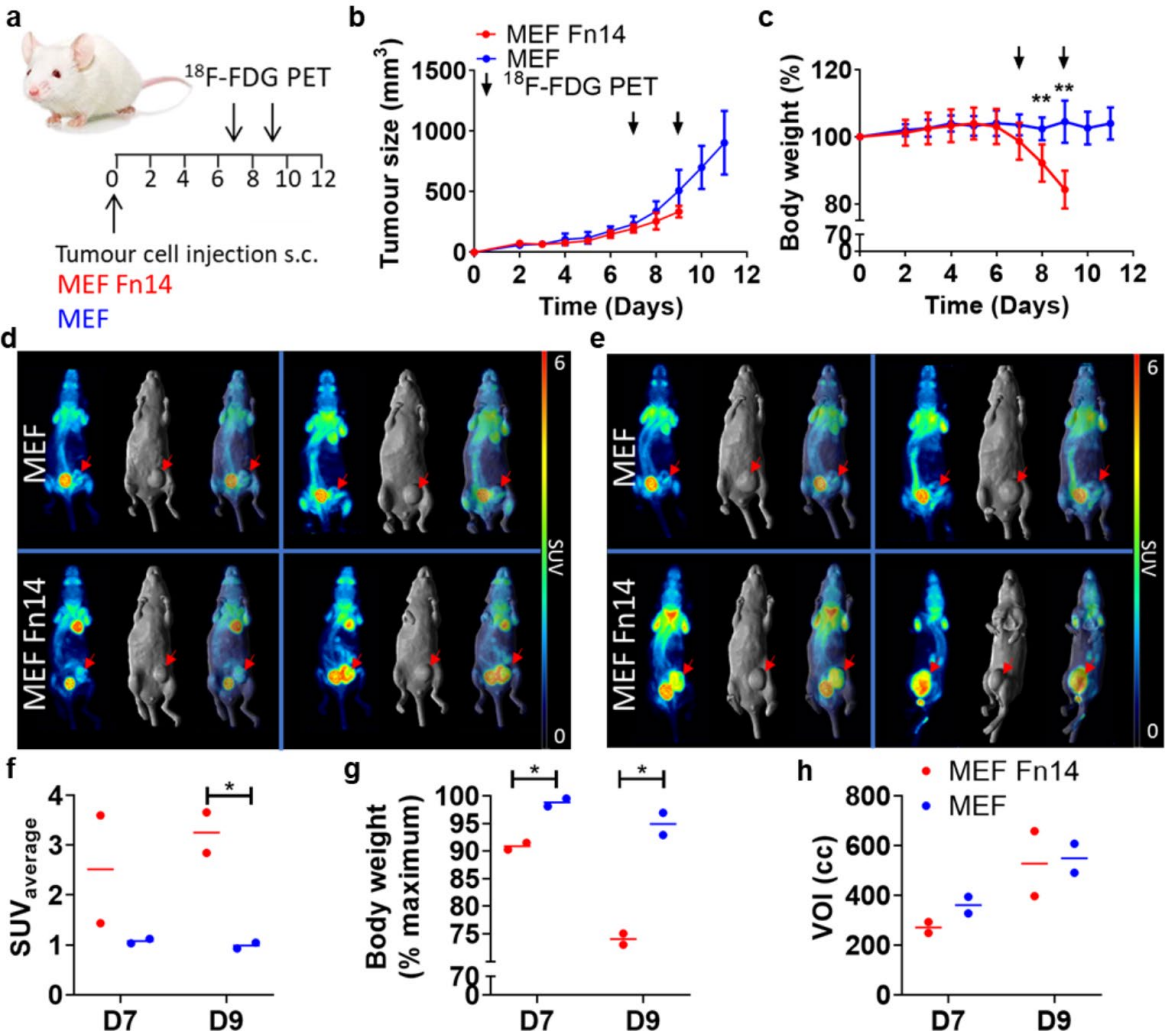
[^18^F]FDG PET imaging in non-cachectic MEF and cachectic MEF Fn14 tumour-bearing mice. **a** Study schematic. **b**, **c** Tumour growth curves (**b**) and body weight curves (**c**) expressed as percentage of body weight at the start of the study of cachexia-inducing MEF Fn14 compared to non-cachexia-inducing MEF tumours in NSG mice; Data presented as mean ± SD, *n* = 6; ** *P* < 0.01. **d**, **e** Whole-body PET (maximum intensity projection), whole-body MR (surface rendered) and fused PET/MR images on day 7 **(d)** and day 9 (**e**) of two mice bearing non-cachexia-inducing MEF (*top panels*) and cachexia-inducing MEF Fn14 (*lower panels*) tumours. Red arrows indicate location of the tumours. **f-h** Quantitative PET analysis. SUV_average_ of [^18^F]FDG tumour uptake (**f**), body weight expressed as maximum body weight (**g**) and tumour volumes (**h**) of imaged mice on day 7 (D7) and day 9 (D9) post cell injection. *n* = 2; *, *P* < 0.05

[^18^F]FDG imaging was performed in cachectic and non-cachectic mice using a mouse embryonic fibroblast cell line MEF H-ras v12 (MEF) that was genetically modified to stably express human Fn14 (MEF Fn14) (Fig. 1a). Six NSG mice were injected with wildtype MEF cells. Another group of six mice were injected with the MEF Fn14 cells. Tumour growth curves of each group of six mice (Fig. 1b) are shown as well as the body weight curves (Fig. 1c). Body weight at each time point is expressed as percentage of body weight at the start of the study. All MEF Fn14 tumour-bearing mice reached the ethical end point of more than 15% body weight loss at 9 days after injection of MEF Fn14 cells. In contrast, the MEF tumour-bearing mice reached the endpoint due to the average tumour size nearing 1000 mm^3^ on day 11.

Two mice of each group were imaged with [^18^F]FDG on day 7 and day 9 post cell injection. Maximum intensity projection PET images, whole-body surface-rendered MRI and PET/MRI overlay images of both mice are shown for day 7 (Fig. 1d) and day 9 (Fig. 1e). PET images demonstrate higher [^18^F]FDG uptake in tumours from cachectic MEF Fn14 mice compared to tumours from non-cachexia inducing MEF mice. Quantitative analysis of tumour uptake is shown in Fig. 1f. Volumes of interest were defined via PET/MRI overlay images. The SUV_average_ was significantly different (*P* < 0.01) and 3-fold higher in the MEF Fn14 tumours compared to the MEF tumours on day 9. Body weight measurements confirmed there was a significant loss of body weight in the imaged MEF Fn14 mice compared to the imaged MEF tumour-bearing mice (body weight (%maximum) ± SD; MEF Fn14, 74.1 ± 1.4; MEF, 94.9 ± 2.8; *P* < 0.05, *n* = 2) (Fig. 1g). The MEF and MEF Fn14 tumours sizes of the imaged mice were comparable at each time point (Fig. 1h), indicating that the increase in [^18^F]FDG is not due to a difference in tumour growth rate.

Individual body weight curves of each mouse used for imaging is shown in Supplementary Figure S1. As body weight loss is evident in mice bearing MEF Fn14 tumours, and body weight is used to calculate SUV values, we also calculated tumour uptake as percentage injected dose per cubic centimeter (%ID/cc) tissue (Suppl. Fig. S2a). This data confirms increased [^18^F]FDG uptake in tumours of cachectic mice. In addition, quantitative PET analysis was also performed for brain, lung and muscle tissue. A significant increase in glucose uptake in the brain was measured, but no significant differences were observed in lung and muscle tissue (Suppl. Fig. S2b-d).

### Targeting Fn14 with mAb 002 prevents cachexia and reduces [^18^F]FDG uptake in C26 tumours

**Fig. 2:**
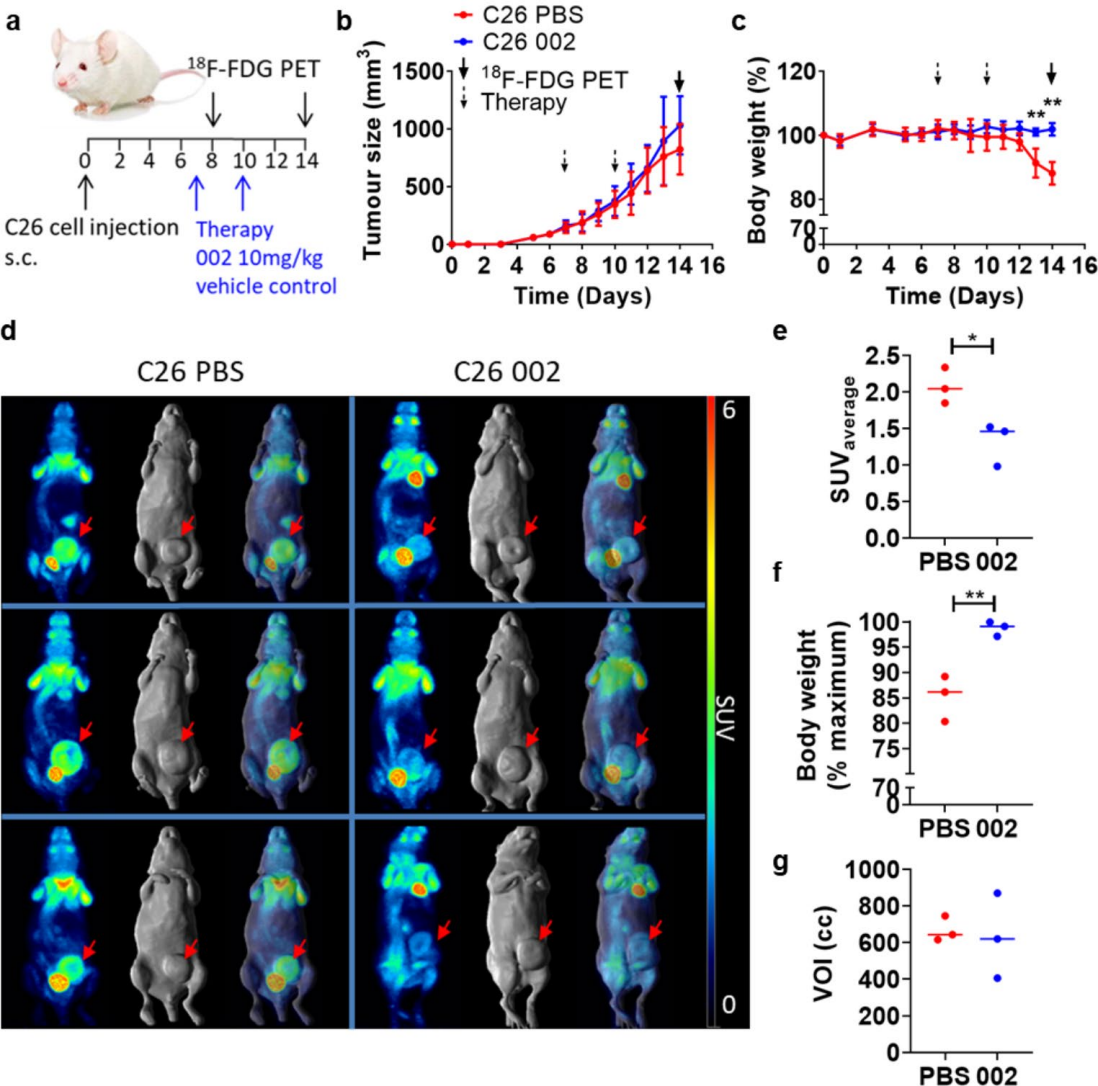
[^18^F]FDG PET imaging in C26 tumour-bearing mice. **a** Study schematic. **b**,** c** Tumour growth curves (**b**) and body weight curves (**c**) expressed as percentage of body weight at the start of the study of cachexia-inducing C26 tumours in NSG mice treated with mAb 002 (10 mg/kg) or vehicle control (PBS) on day 7 and day 10 post cell injection; data presented as mean ± SD, *n* = 5; ** *P* < 0.01. **d** Whole-body PET (maximum intensity projection), whole-body MR (surface rendered) and fused PET/MR images on day 14 of three per treatment group (PBS, left column; mAb 002, right column). Red arrows indicate location of the tumours. **e-g** Quantitative PET analysis. SUV_average_ of [^18^F]FDG tumour uptake (**e**), body weight expressed as percentage of maximum body weight (**f**) and tumour volumes (**g**) of imaged mice on day 14 post cell injection. *n* = 3; *, *P* < 0.05; **, *P* < 0.01

In a second study, [^18^F]FDG imaging was performed in mice bearing the cachexia-inducing colon tumour cell line (C26). One group of mice was treated with the murine 002 anti-Fn14 antibody and the second group received the vehicle control (Fig. 2a). Tumour growth curves (Fig. 2b) of each group are shown as well as the body weight curves (Fig. 2c). In this model, body weight loss is expected to start around 11 days after injection of tumour cells. At day 14, mice treated with vehicle control showed significant body weight loss compared to mice treated with the 002 antibody (*P* < 0.01, *n* = 3) (Fig. 2c). There was no significant difference in tumour growth between the two groups (*P* = 0.2063, *n* = 3) (Fig. 2b).

Three mice of each group were selected for [^18^F]FDG imaging. Mice were imaged on day 8 and day 14 post tumour cell injection. On day 8, no significant difference in [^18^F]FDG uptake by the tumour and no significant body weight loss was observed between the groups (data not shown). Figure 2d shows the results of [^18^F]FDG imaging on day 14. Mice in the left panels were treated with vehicle control and show a higher tumour uptake of [^18^F]FDG compared to the mAb 002 treated mice shown in the panels on the right. Quantitative PET analysis was performed for tumour uptake (Fig. 2e). The SUV_average_ was significantly (*P* < 0.05) reduced for mice treated with the 002 antibody (SUV_average_, 1.32 ± 0.30) compared to PBS treated mice (SUV_average_, 2.08 ± 0.25). Imaged mice treated with PBS showed significant (*P* < 0.01) body weight loss (body weight (%maximum), 85.22 ± 4.50) compared to mAb 002 treated mice (body weight (% maximum), 98.71 ± 1.43) (Fig. 2f). Again, tumour sizes of the imaged mice were comparable, indicating that the difference in [^18^F]FDG uptake is not due to a difference in tumour growth rate (*P* = 0.806, *n* = 3) (Fig. 2g). These results demonstrate that anti-Fn14 therapy prevents increase in [^18^F]FDG uptake in cachexia-inducing C26 tumours and this coincides with mice maintaining their body weight.

Individual body weight curves of each mouse used for imaging are shown in Supplementary Figure S3. Tumour uptake calculated as percentage injected dose per cubic centimeter (%ID/cc) of tissue (Suppl. Fig. S4a) confirms reduced [^18^F]FDG uptake in tumours of C26-tumour-bearing mice treated with mAb 002. Quantitative PET analysis of brain, lung and muscle tissue showed a small but not significant increase in brain uptake (Suppl. Fig. S2b). Similar to the MEF Fn14 model, no differences in glucose uptake was observed in lung and muscle tissue (Suppl. Fig. S4c, d).

### [^18^F]FDG uptake in cachexia-inducing C26 tumours compared to C26 Fn14 knock-out tumours

**Fig. 3:**
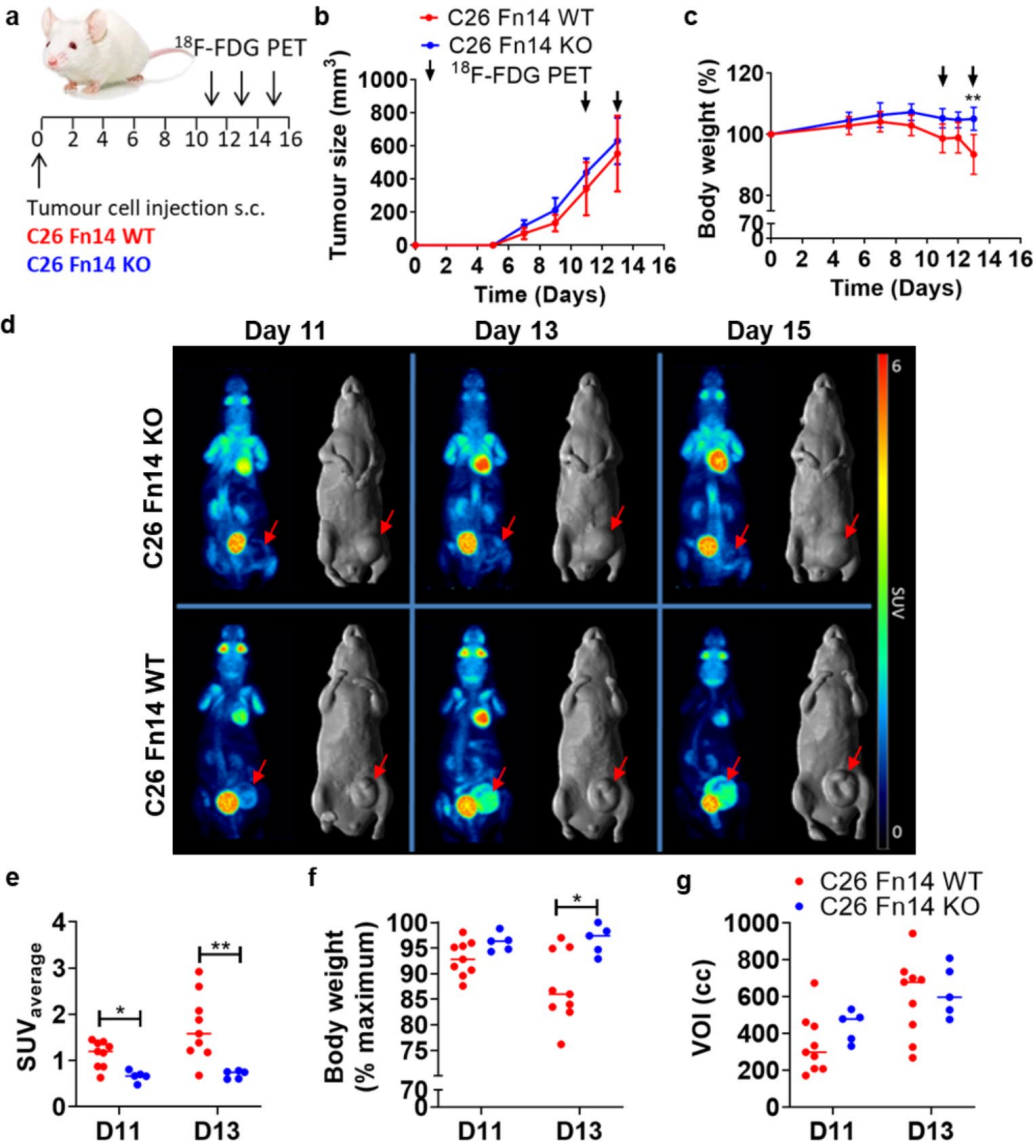
[^18^F]FDG PET imaging in C26 Fn14 wildtype (cachectic) and C26 Fn14 knock-out (non-cachectic) tumour-bearing NSG mice. **a** Study schematic. **b**, **c** Tumour growth curves **(b)** and body weight curves (**c**) expressed as percentage of body weight at the start of the study; data presented as mean ± SD; C26 Fn14 WT, *n* = 9; C26 Fn14 KO, *n* = 5; ** *P* < 0.01. **d** Whole-body PET (maximum intensity projection) and whole-body MR (surface rendered) images of one representative mouse of C26 Fn14 KO (*top*) and C26 Fn14 WT (*bottom*) on day 11, 13 and 15 post cell injection. Red arrows indicate location of the tumours. **e-g** Quantitative PET analysis. SUV_average_ of [^18^F]FDG tumour uptake (**e**), body weight expressed as percentage of maximum body weight (**f**) and tumour volumes (**g**) of imaged mice on day 11 and 13 post cell injection. *, *P* < 0.05; **, *P* < 0.01

To further confirm that Fn14 receptor activation is linked to the glucose metabolism of cachexia-inducing tumours, we investigated [^18^F]FDG imaging in C26 wildtype tumours (C26 Fn14 WT) expressing Fn14 versus C26 Fn14 knock-out tumours (C26 Fn14 KO) (Fig. 3a). The tumour growth and body weight curves summarize the results of two independent experiments. In total, nine C26 Fn14 WT and five C26 Fn14 KO tumour-bearing mice were imaged. All mice were imaged on day 11 and day 13. When possible and mice had not reached ethical endpoint (i.e. tumour size ≥ 1000 mm^3^ or body weight loss ≥ 15% of maximum body weight), mice were imaged again on day 15.

There was no significant difference in tumour growth between C26 Fn14 WT and C26 Fn14 KO tumours (tumour volume on day 13 (mm^3^ ± SD); C26 Fn14 WT, 554 ± 228, *n* = 9; C26 Fn14 KO, 629 ± 140, *n* = 5; *P* = 0.5206) (Fig. 3b). Significant body weight loss (P ˂ 0.05) was reached on day 13 in the C26 Fn14 WT group (body weight (% maximum ± SD), 87.33 ± 6.96), whilst the C26 Fn14 KO group maintained their body weight (96.66 ± 2.85). One representative mouse is shown of each group imaged on day 11, 13 and 15 (Fig. 3d). As most of the C26 Fn14 WT mice had reached endpoint by day 14, the quantitative PET analysis was only performed on the images obtained on day 11 and day 13. In non-cachectic mice bearing C26 Fn14 KO tumours, [^18^F]FDG tumour uptake was significantly lower (*P* < 0.01) (SUV_average_, 0.70 ± 0.09; % maximum body weight, 96.66 ± 0.85; *n* = 5) than in cachectic mice bearing C26 Fn14 WT tumours (SUV_average_, 1.73 ± 0.72; % maximum body weight, 87.33 ± 6.96; n = *9*) (Fig. 3e-f).

Individual body weight curves of each mouse are shown in Supplementary Figure S5. Tumour uptake calculated as percentage injected dose per cubic centimeter (%ID/cc) of tissue (Suppl. Fig. S6a) confirms significantly higher [^18^F]FDG uptake in C26 Fn14 WT tumours compared to C26 Fn14 KO tumours. A significant higher brain uptake of [^18^F]FDG was observed by day 13 in C26 Fn14 WT tumours compared to C26 Fn14 KO tumours (Suppl. Fig. S6b). No differences in glucose uptake were observed in lung and muscle tissue (Suppl. Fig. S6c, d).

### Quantitative analysis of l-lactate in cachexia-inducing versus non-cachexia-inducing tumours

**Fig. 4:**
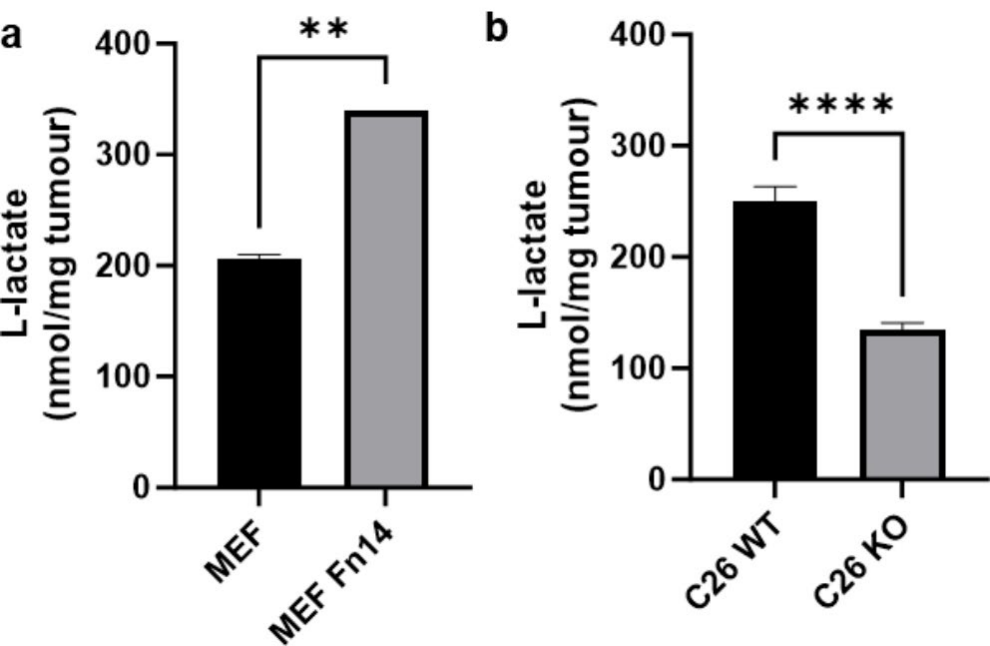
Quantitation of l-lactate in cachexia-inducing versus non-cachexia-inducing tumours used for [^18^F]FDG imaging. **a** L-lactate in MEF and MEF Fn14 tumours (*n* = 2, ** *P* < 0.005). **b** L-lactate in C26 WT and C26 Fn14 KO tumours (*n* = 5, **** *P* < 0.0001). All data are presented as mean ± SEM

As a by-product of glucose metabolism, l-lactate production should also be increased if there is indeed a rise in levels of glucose metabolism. To assess this, l-lactate was measured in tumours used for [^18^F]FDG imaging. There was an increase in lactate production (nmol/mg tumour ± SEM) in cachexia-inducing MEF Fn14 tumours (340.0 ± 0.0, *n* = 2) compared to non-cachexia-inducing MEF tumours (205.4 ± 4.6, *n* = 2; *P* < 0.01) (Fig. 4a). In contrast, when Fn14 is knocked out, C26 Fn14 KO tumours (134.1 ± 6.9, *n* = 5) produce significantly less lactate compared to their C26 Fn14 WT counterparts (250.1 ± 13.4, *n* = 5; *P* < 0.0001) (Fig. 4b), suggestive of altered glucose metabolism and downstream lactate production caused by expression of Fn14 in tumours.

## Discussion

[^18^F]FDG is a marker for glucose metabolism. [^18^F]FDG PET is frequently used in neurology, cardiology and oncology including application for diagnosis, staging, and monitoring treatment of cancers [18, 19]. As cachexia is a syndrome involving metabolism and a complication of cancer, the metabolic changes occurring in cachectic versus non-cachectic patients or monitoring treatment of cachexia may be able to be distinguished by whole body molecular imaging with [^18^F]FDG. In this study we used mouse models of cachexia to test the hypothesis that cancer cahexia may be associated with changes in tumour glucose metabolism, and that targeting Fn14 signalling in tumour, which is associated with body weight loss, can alter the [^18^F]FDG tumour uptake. This may also potentially be used to help with selection of cancer cachexia patients that might benefit from anti-Fn14 targeting therapy, such as with antibody 002.

Utilising whole body [^18^F]FDG PET imaging, an increase of glucose uptake over time was observed in tumours and brain of cachexia-inducing versus non-cachexia-inducing tumour-bearing mice, suggesting a higher glucose metabolic activity in tumours of cachectic mice. Targeting Fn14 with mAb 002 or by genetic deletion was able to prevent the increased uptake of [^18^F]FDG in C26, demonstrating that the Fn14 receptor is linked to the glucose metabolic pathway of tumours. Our data supports previous findings by Penet et al. [20], that cachexia-inducing tumours show an increase in [^18^F]FDG uptake in tumour and brain tissue, however, our study highlights the role of the Fn14 receptor in regulating [^18^F]FDG uptake. In contrast to Penet et al. [20], our models did not show significant differences in [^18^F]FDG uptake in lung tissue.

The tumour microenvironment (TME) and the tumour are known to be metabolically distinct from normal tissues, and this environment may drive cancer-cachexia specific Fn14 pathways. Cancer cells have adapted via many alterations to promote survival, growth, migration, invasion, and metastasis, one of the most distinctive is the ability to metabolise glucose to lactate [16, 17]. This appears to be advantageous to cell growth despite less ATP is generated per molecule of glucose compared to oxidative phosphorylation [16]. It has been proposed that the TME may directly contribute [22, 23] to tumour cell metabolism by limiting the availability of glucose and creating an environment of nutrient competition in surrounding cells and triggering a hypoxic state [24, 25]. Hypoxia corresponds with a decrease in ATP generation through oxidative phosphorylation and an increase in glycolysis [26, 27]. Our results suggest for the first time a role for Fn14 in altered glycolytic capacity of tumour cells. When human Fn14 is expressed on MEF tumour cells, there is an increase in lactate production through the reduction of pyruvate to lactate. This is further supported by the reduction of lactate in C26 KO tumours in comparison to C26 WT tumours, directly implicating Fn14 in altered glucose uptake and lactate metabolism in cancer cachexia-inducing tumour models.

In conclusion, our results demonstrate that the Fn14 receptor activation is linked to glucose metabolism of cachexia-inducing tumours. A clinical trial with [^18^F]FDG PET in cachectic patients is currently ongoing (NCT04127981) to further evaluate this observation in cancer patients.

## Electronic supplementary material

Below is the link to the electronic supplementary material.

Supplementary Material 1

## Data Availability

The datasets generated during and/or analysed during the current study are available from the corresponding author on reasonable request.

## References

[1] Hopkinson JB, Wright DN, McDonald JW, Corner JL. The prevalence of concern about weight loss and change in eating habits in people with advanced cancer. J Pain Symptom Manage. 2006;32:322–31. 17000349 10.1016/j.jpainsymman.2006.05.012

[2] Poole K, Froggatt K. Loss of weight and loss of appetite in advanced cancer: a problem for the patient, the carer, or the health professional? Palliat Med. 2002;16:499–506. 12465697 10.1191/0269216302pm593oa

[3] Schmidt SF, Rohm M, Herzig S, Berriel Diaz M. Cancer Cachexia: more than skeletal muscle wasting. Trends Cancer. 2018;4:849–60. 30470306 10.1016/j.trecan.2018.10.001

[4] Evans WJ, Morley JE, Argiles J, Bales C, Baracos V, Guttridge D, et al. Cachexia: a new definition. Clin Nutr. 2008;27:793–9. 18718696 10.1016/j.clnu.2008.06.013

[5] Rowland KM Jr., Loprinzi CL, Shaw EG, Maksymiuk AW, Kuross SA, Jung SH, et al. Randomized double-blind placebo-controlled trial of cisplatin and etoposide plus megestrol acetate/placebo in extensive-stage small-cell lung cancer: a North Central Cancer Treatment Group study. J Clin Oncol. 1996;14:135–41. 8558188 10.1200/JCO.1996.14.1.135

[6] Lok C. Cachexia: the last illness. Nature. 2015;528:182–3. 26659165 10.1038/528182a

[7] Cao Z, Zhao K, Jose I, Hoogenraad NJ, Osellame LD. Biomarkers for Cancer Cachexia: a Mini Review. Int J Mol Sci. 2021; 22. 10.3390/ijms22094501 PMC8123431 33925872

[8] Burkly LC, Michaelson JS, Zheng TS. TWEAK/Fn14 pathway: an immunological switch for shaping tissue responses. Immunol Rev. 2011;244:99–114. 22017434 10.1111/j.1600-065X.2011.01054.x

[9] Winkles JA. The TWEAK-Fn14 cytokine-receptor axis: discovery, biology and therapeutic targeting. Nat Rev Drug Discov. 2008;7:411–25. 18404150 10.1038/nrd2488 PMC3018765

[10] Lynch CN, Wang YC, Lund JK, Chen YW, Leal JA, Wiley SR. TWEAK induces angiogenesis and proliferation of endothelial cells. J Biol Chem. 1999;274:8455–9. 10085077 10.1074/jbc.274.13.8455

[11] Jakubowski A, Ambrose C, Parr M, Lincecum JM, Wang MZ, Zheng TS, et al. TWEAK induces liver progenitor cell proliferation. J Clin Invest. 2005;115:2330–40. 16110324 10.1172/JCI23486 PMC1187931

[12] Vince JE, Chau D, Callus B, Wong WW, Hawkins CJ, Schneider P, et al. TWEAK-FN14 signaling induces lysosomal degradation of a cIAP1-TRAF2 complex to sensitize tumor cells to TNFalpha. J Cell Biol. 2008;182:171–84. 18606850 10.1083/jcb.200801010 PMC2447903

[13] Tornatore L, Thotakura AK, Bennett J, Moretti M, Franzoso G. The nuclear factor kappa B signaling pathway: integrating metabolism with inflammation. Trends Cell Biol. 2012;22:557–66. 22995730 10.1016/j.tcb.2012.08.001

[14] Hoesel B, Schmid JA. The complexity of NF-kappaB signaling in inflammation and cancer. Mol Cancer. 2013;12:86. 23915189 10.1186/1476-4598-12-86 PMC3750319

[15] Johnston AJ, Murphy KT, Jenkinson L, Laine D, Emmrich K, Faou P, et al. Targeting of Fn14 prevents Cancer-Induced Cachexia and Prolongs Survival. Cell. 2015;162:1365–78. 26359988 10.1016/j.cell.2015.08.031

[16] Osellame LD, Blacker TS, Duchen MR. Cellular and molecular mechanisms of mitochondrial function. Best Pract Res Clin Endocrinol Metab. 2012;26:711–23. 23168274 10.1016/j.beem.2012.05.003 PMC3513836

[17] Rich P. Chemiosmotic coupling: the cost of living. Nature. 2003;421:583. 12571574 10.1038/421583a

[18] Kiamanesh Z, Ayati N, Sadeghi R, Hawkes E, Lee ST, Scott AM. The value of FDG PET/CT imaging in outcome prediction and response assessment of lymphoma patients treated with immunotherapy: a meta-analysis and systematic review. Eur J Nucl Med Mol Imaging. 2022;49:4661–76. 35932329 10.1007/s00259-022-05918-2 PMC9606078

[19] Murphy PS, Galette P, van der Aart J, Janiczek RL, Patel N, Brown A. The role of clinical imaging in oncology drug development: progress and new challenges. Br J Radiol. 2023: 20211126. 10.1259/bjr.20211126 PMC10546429 37393537

[20] Penet MF, Gadiya MM, Krishnamachary B, Nimmagadda S, Pomper MG, Artemov D, et al. Metabolic signatures imaged in cancer-induced cachexia. Cancer Res. 2011;71:6948–56. 21948967 10.1158/0008-5472.CAN-11-1095 PMC3217079

[21] Olaechea S, Gannavarapu BS, Alvarez C, Gilmore A, Sarver B, Xie D, et al. Primary tumor Fluorine-18 fluorodeoxydglucose ((18)F-FDG) is Associated with Cancer-Associated Weight loss in Non-small Cell Lung Cancer (NSCLC) and portends worse survival. Front Oncol. 2022;12:900712. 35814438 10.3389/fonc.2022.900712 PMC9263563

[22] Hsu PP, Sabatini DM. Cancer cell metabolism: Warburg and beyond. Cell. 2008;134:703–7. 18775299 10.1016/j.cell.2008.08.021

[23] Kim JW, Dang CV. Cancer’s molecular sweet tooth and the Warburg effect. Cancer Res. 2006;66:8927–30. 16982728 10.1158/0008-5472.CAN-06-1501

[24] Chang CH, Qiu J, O’Sullivan D, Buck MD, Noguchi T, Curtis JD, et al. Metabolic competition in the Tumor Microenvironment is a driver of Cancer Progression. Cell. 2015;162:1229–41. 26321679 10.1016/j.cell.2015.08.016 PMC4864363

[25] Ho PC, Bihuniak JD, Macintyre AN, Staron M, Liu X, Amezquita R, et al. Phosphoenolpyruvate is a metabolic checkpoint of anti-tumor T cell responses. Cell. 2015;162:1217–28. 26321681 10.1016/j.cell.2015.08.012 PMC4567953

[26] Vyas S, Zaganjor E, Haigis MC. Mitochondria Cancer Cell. 2016;166:555–66. 10.1016/j.cell.2016.07.002 PMC5036969 27471965

[27] Zong WX, Rabinowitz JD, White E. Mitochondria and Cancer. Mol Cell. 2016;61:667–76. 26942671 10.1016/j.molcel.2016.02.011 PMC4779192

