# Supplementary material for: Targeting Fn14 as a therapeutic target for cachexia reprograms the glycolytic pathway in tumour and brain in mice
Source: Eur J Nucl Med Mol Imaging. 2024 Jul 26;51(13):3854–62. doi: 10.1007/s00259-024-06836-1 (PMC11527931; doi:10.1007/s00259-024-06836-1)
Supplement: Supplementary file 1 — Supplementary Material 1 [file 259_2024_6836_MOESM1_ESM.docx]

**Targeting Fn14 as a therapeutic target for cachexia reprograms the glycolytic pathway in tumour and brain in ~~tumour-bearing~~ mice**

Ingrid Julienne Georgette Burvenich^1,2^, Laura Danielle Osellame^1,2,3^, Angela Rigopoulos^1^, Nhi Huynh^1,2^, Zhipeng Cao^1,2,4^, Nicholas Johannes Hoogenraad^3^, and Andrew Mark Scott^1, 2, 3,4, 5^

^1^Tumour Targeting Laboratory, Olivia Newton-John Cancer Research Institute, Melbourne, VIC 3084, Australia

^2^School of Cancer Medicine, La Trobe University, Melbourne, VIC 3086, Australia

^3^Department of Biochemistry and Genetics, La Trobe Institute for Molecular Science, La Trobe University, Melbourne, VIC 3083, Australia

^4^Department of Molecular Imaging and Therapy, Austin Health, Melbourne, VIC 3083, Australia

^5^Department of Medicine, University of Melbourne, Melbourne, VIC 3052, Australia

**Corresponding Author:**

Professor Andrew M. Scott,

Tumour Targeting Laboratory, Olivia Newton-John Cancer Research Institute,

145 Studley Road, Heidelberg, Victoria 3084, Australia

Phone: 61-39496-5876; Fax: 61-39496-5334;

E-mail: andrew.scott@onjcri.org.au

**Short Title**: [^18^F]FDG imaging in Fn14 driven cancer cachexia

**INDEX**

**Supplementary Figures S1, S2, S3, S4, S5, S6**

**
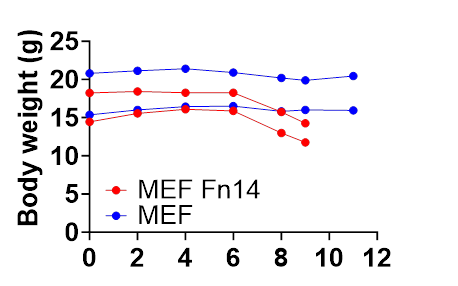
**

**Suppl. Fig. S1** Individual body weight curves of cachectic MEF Fn14 (*red*) and non-cachectic MEF (*blue*) tumour-bearing mice used for [^18^F]FDG imaging.

**
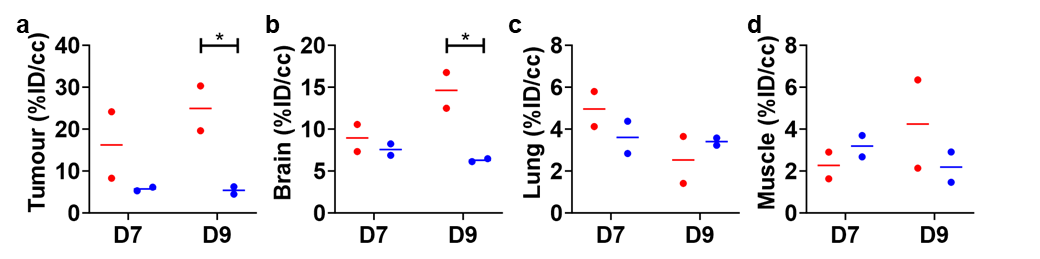
**

**Suppl. Fig. S2** Quantitative PET analysis of [^18^F]FDG imaging in non-cachectic MEF (*blue*) and cachectic MEF Fn14 (*red*) tumour-bearing mice. [^18^F]FDG tumour uptake **(a)**, brain uptake **(b)**, lung uptake **(c)** and muscle uptake **(d)** of imaged mice on day 7 (D7) and day 9 (D9) post cell injection expressed as percentage injected dose per cubic centimeter of tissue (%ID/cc). *n* = 2; *, *P* < 0.05. Statistical text used was a one-tailed unpaired t test.

**
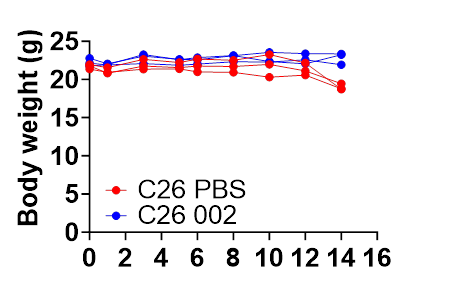
**

**Suppl. Fig. S3** Individual body weight curves of C26 tumour-bearing mice treated with mAb 002 (10 mg/kg, *blue*) or vehicle control (PBS, *red*) used for [^18^F]FDG imaging.

**
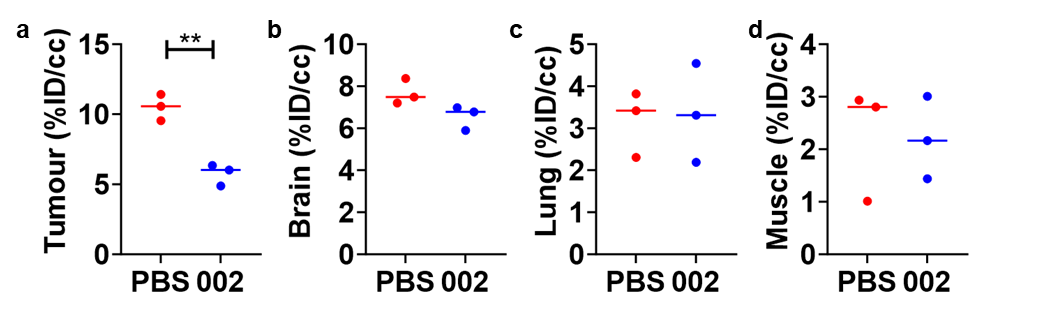
**

**Suppl. Fig. S4** Quantitative PET analysis of [^18^F]FDG imaging in C26 tumour-bearing mice treated with mAb 002 (10 mg/kg) or vehicle control (PBS) on day 7 and day 10 post cell injection. [^18^F]FDG tumour uptake **(a)**, brain uptake **(b)**, lung uptake **(c)** and muscle uptake **(d)** of imaged mice on day 14 post cell injection expressed as percentage injected dose per cubic centimeter of tissue (%ID/cc). *n* = 3; **, *P* < 0.01. Statistical test used was a two-tailed unpaired t test.

**
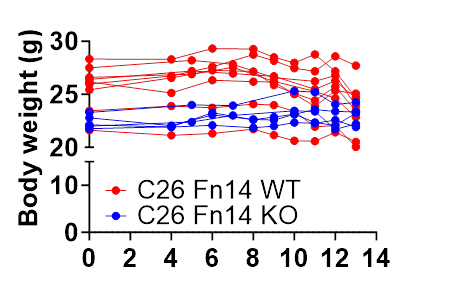
**

**Suppl. Fig. S5** Individual body weight curves of C26 Fn14 WT (*red*) and C26 Fn14 KO (*blue*) tumour-bearing mice used for [^18^F]FDG imaging.

**
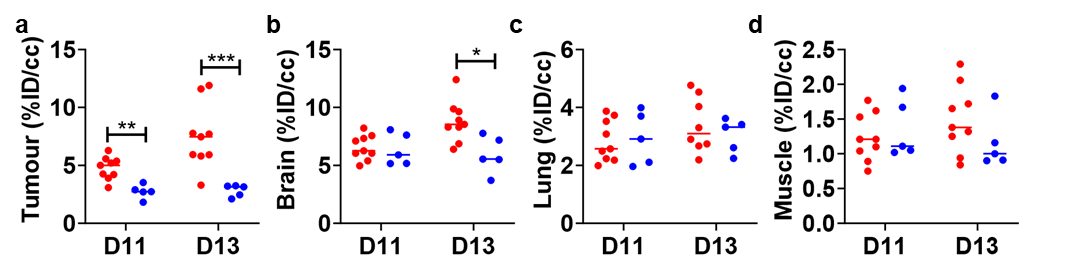
**

**Suppl. Fig. S6** Quantitative PET analysis of [^18^F]FDG imaging in C26 Fn14 wildtype (cachectic, *red*) and C26 Fn14 knock-out (non-cachectic, *blue*) tumour-bearing NSG mice on day 11 (D11) and day 13 (D13) post cell injection. [^18^F]FDG tumour uptake **(a)**, brain uptake **(b)**, lung uptake **(c)** and muscle uptake **(d)** of imaged mice expressed as percentage injected dose per cubic centimeter of tissue (%ID/cc). C26 Fn14 WT, *n* = 9; C26 Fn14 KO, *n* = 5; *, *P* = 0.011; **, P = 0.002; ***, *P* = 0.001. Statistical test used was a two-tailed Mann Whitney test.
